# TREATMENT ADHERENCE AMONG CHILDREN AND ADOLESCENTS IN A CYSTIC FIBROSIS REFERENCE CENTER

**DOI:** 10.1590/1984-0462/2020/38/2018338

**Published:** 2020-06-05

**Authors:** Bianca Sampaio Bonfim, Valmir Machado de Melo, Fernanda Matos Fontenelle, Edna Lúcia Souza

**Affiliations:** a Universidade Federal da Bahia, Salvador, BA, Brazil.

**Keywords:** Cystic fibrosis, Medication adherence, Patient compliance, Pediatrics, Therapeutics, Fibrose cística, Adesão à medicação, Cooperação do paciente, Pediatria, Terapêutica

## Abstract

**Objective:**

To evaluate the level of self-referenced treatment adherence (TA) and its association with clinical and sociodemographic variables in patients with cystic fibrosis assisted at a reference center, as well as compare the level of self-referenced TA with that presumed by the multidisciplinary team.

**Methods:**

This is a cross-sectional study that included children and adolescents aged between 0-20 years with cystic fibrosis. Adolescents older than 14 years or their guardians, when younger than 14 years old, were interviewed using a standardized questionnaire. Professionals from the multidisciplinary clinic filled out another form with their impressions of the patients’ TA. Clinical and laboratory data were obtained in the medical records. The TA was considered satisfactory if the total adherence index (TAI) was equal or higher than 80%.

**Results:**

53 patients were included with a median age of 112 months. The mean TAI was 83.2%. The mean TAIs for dornase alfa, pancreatic enzymes, continued use of inhaled tobramycin, vitamins supplements, nutritional supplements and dietary orientation was respectively: 86.1; 96.6; 78.6; 88.1; 51.8 and 78%. Children younger than 14 years presented better TA (p=0.021). The correlation between the self-referenced TA and the one presumed by the multidisciplinary team ranged from 0,117 to 0.402, being higher for Psychology and Nutrition professionals.

**Conclusions:**

The TAI was high particularly among children younger than 14 years. There was a positive correlation between the self-referenced TA and the one presumed by the Psychology (p=0.032) and the nutrition (p=0.012) professionals.

## RESUMO

**Objetivo:**

Avaliar o grau de adesão ao tratamento (AT) autorreferida e pesquisar sua associação com variáveis clínicas e sociodemográficas em pacientes com fibrose cística (FC) de um centro de referência e comparar o grau de AT autorreferida com o presumido pela equipe multidisciplinar.

**Métodos:**

Estudo transversal, incluindo crianças e adolescentes com FC entre zero e 20 anos. Foram realizadas entrevistas com os adolescentes maiores de 14 anos ou com os responsáveis dos mais jovens, utilizando-se formulário padronizado. Profissionais do centro preencheram outro formulário com suas impressões da AT dos pacientes. Registraram-se dados clínicos por meio da revisão de prontuários. A AT foi considerada satisfatória se o índice de adesão global (IAG) fosse igual ou superior a 80%.

**Resultados:**

Foram incluídos 53 pacientes, com mediana de idade de 112 meses. O IAG médio foi de 83,2%. Os índices de adesão à terapêutica (IAT) médios para alfadornase, enzimas pancreáticas, tobramicina inalatória de uso contínuo, suplementos vitamínicos, suplementos nutricionais e orientações dietéticas foram, respectivamente, 86,1; 96,6; 78,6; 88,1; 51,8 e 78%. Crianças com idade inferior a 14 anos apresentaram melhor AT (p=0,021). As correlações entre a AT autorreferida e a presumida pelos profissionais variaram de 0,117 a 0,402, sendo maiores para a psicologia e a nutrição.

**Conclusões:**

A taxa global de AT foi elevada, principalmente para menores de 14 anos. Houve correlação positiva entre a AT autorreferida e as avaliações da psicologia (p=0,032) e da nutrição (p=0,012).

## INTRODUCTION

Cystic fibrosis (CF) is the most common genetic disease in Caucasians, being characterized by multisystem clinical manifestations and requiring a complex therapeutic regimen, which may include the use of bronchodilators, mucolytics, antibiotics, vitamin supplements, enzyme replacement therapy (ETR), insulin and, more recently, protein modulators of Cystic Fibrosis Transmembrane Conductance Regulator (CFTR). In addition, dietary specifications and daily respiratory physical therapy sessions are required.^1^ ^,^ ^2^ ^,^ ^3^ The therapeutic regimen of these patients has, on average, seven components and require 108 to 180 minutes per day,^4^ ^,^ ^5^ which makes treatment adherence (TA) difficult. The World Health Organization (WHO) defines TA as the extent to which an individual’s behavior (regarding medication use, adherence to diet, lifestyle changes etc.) coincides with the recommendation agreed between them and the health professional.^6^

Overall, absence of TA has a major impact on health costs,^7^ which may reach US$ 100 billion annually for the United States, accounting for 10% of hospital admissions and 23% of total home admissions.^8^ Noncompliance in CF also contributes to increase in hospital stay, increased occurrence of pulmonary exacerbations, and loss of pulmonary function - the leading cause of premature death related to the disease.^9^ The overall rate of TA in CF varies from 31 to 79%.^7^ ^,^ ^10^ Multiple factors potentially influencing TA have been reported in the literature, such as parental supervision, severity of disease, bond with the health team, age, perceived repercussions of non-adherence, priorities competing with adherence, gender, forgetfulness, privacy concerns,^11^ ^,^ ^12^ ^,^ ^13^ ^,^ ^14^ among others.

A thorough understanding of TA and related factors requires studying diverse populations. In Brazil, studies on TA in CF are scarce, with a percentage of high-TA patients ranging from 59 to 81.6%.^15^ ^,^ ^16^ ^,^ ^17^ Therefore, the present study has the following objectives: to determine the degree of self-reported TA related to medication, respiratory physical therapy, and the diet recommended for CF patients from a referral center; to evaluate how TA varies according to clinical and sociodemographic data; and to compare the degree of compliance reported by patients with that assumed by the multidisciplinary team.

## METHOD

A cross-sectional study was conducted with patients (aged zero to 20 years old) assisted at a pediatric referral center for CF treatment. Patients are treated, on average, every three months, by a team of physicians and professionals in the areas of physical therapy, nutrition, pharmacy, social assistance, and psychology, aiming to develop individualized and integral care, which enables a close contact with patient, encouraging health promotion in this context. Some of the regularly medications (dornase alfa, pancreatic enzymes, and inhaled tobramycin) prescribed for CF are made available by the Unified Health System (SUS) and handed to patients at the center itself after each routine consultation. Thus, patients do not travel or have additional costs to access medication.

Data were collected during routine outpatient consultations in interviews conducted by the same researcher, in a private place, protecting patient privacy, from February 2015 to August 2016. Forms were filled out with variables possibly related to TA (available with corresponding author), questions about medication use, and socioeconomic data. For those under 14 years old, the questions were addressed to their guardians, while those over 14 were interviewed directly. The evaluation of each patient’s TA was also requested by professional of physical therapy, nutrition, pharmacy, psychology and pneumology (forms available with corresponding author), giving them a score from one (minimum TA) to ten (maximum TA), based on their impressions when seeing the participant.

Initially, the questionnaires were randomly applied to 12 patients in order to verify their practical feasibility and identify the main adjustments to be made (pilot study). Due to the low level of understanding by patients, we opted to interview the adolescents or guardians, depending on the age group.

In terms of casuistry, all children and adolescents aged zero to 20 years with a confirmed diagnosis of CF were included by two positive sweat tests (Chlorine ≥60 mEq/L) and/or identification of two pathological CFTR gene mutations, and also six patients with CF-compatible clinic and questionable sweat test, who were regularly taking CF treatment medications. All patients should be followed up for at least six months at the referral center. Exclusion criteria were withdrawal from participation in the study and absence of continuous use of specific CF medications. After applying these criteria, the final sample was constituted of 53 patients. This project was approved by the Research Ethics Committee of the institution in which it was conducted, Opinion No. 843.869, of October 23, 2014. Free and informed consent forms were obtained from the guardians or patients, depending on the age group, and terms of assent, where relevant.

The different treatment protocols were evaluated, seeking to identify differences in adherence rates between them. During the interviews, information regarding the patients’ socioeconomic conditions was obtained, as well as the difficulties they reported facing in adhering to treatment.

During review of medical records, the following clinical data were collected:

- Forced expiratory volume in the first second (FEV_1_) of the best spirometry of the year of the interview or most recent of each patient eligible for the exam.
- Most recent anthropometric data, dating closest to the interview day (body mass index - BMI, expressed in kg/m^2^), plus Z scores for BMI/age (patients older than 5 years) and weight/height Z score (under five).
- Shwachman-Kulczycki score of the same year of interview or most recent. This score is an instrument for clinical and functional assessment of CF patients, divided into four categories - general activity, physical examination, nutrition and radiological findings - with a final score ranging from 20 to 100 points.^18^
- Hospital cycles of intravenous antibiotics in the past two years.
- Age of patients at diagnosis.

The degree of TA was calculated using the Therapy Adherence Index (TAI), obtained for each of the treatment protocols used by the patient through the following operation: TAI=frequency of use by the patient/standard frequency of use (x100), where the frequencies used by patients corresponded to the following self-reported data during interviews with them:

- Number of times of inhaled dornase alfa administration per week.
- Total meals using pancreatic enzymes per day.
- Number of meals in which oil or olive oil is used per day.
- Number of spoons of nutritional supplements per day.
- Number of drops of the vitamin supplement used per day.
- Inhaled Tobramycin: total inhaled ampoules on alternate months for patients receiving continuous antibiotic therapy every other month.

The overall adherence index (OAI) was calculated as the arithmetic mean of the percentages obtained for each treatment protocol. TA was considered satisfactory for patients with OAI from 80% and unsatisfactory below this percentage. The AT (satisfactory or unsatisfactory) was then compared with the estimated adherence scores indicated by the health professionals.

The data obtained were recorded in a standard form and stored in a database in the Statistical Package for the Social Sciences (SPSS), version 20 (SPSS^®^, Chicago, IL, USA). Descriptive data were expressed as simple and relative frequencies of the studied qualitative variables, as well as means, standard deviation (SD), median or minimum and maximum values for quantitative variables. Income was expressed as median and interquartile range. Continuous variables with normal distribution were expressed as means and SD; and those with non-normal distribution, as median and interquartile range. The normality of numerical variables was verified by descriptive statistics, graphical analysis and the Shapiro-Wilk test.

For the association between adherence (satisfactory and unsatisfactory) and sociodemographic and clinical data (categorical variables), the chi-square test was used, assuming the presence of association if p<0.05. For the correlation between OAI and TA perception scores of different professionals (pharmacy, psychology, physiotherapy, pediatric pneumology, and nutrition), the Spearman’s correlation was used. Correlations were classified according to the following criteria:

- between 1.00 and 0.90 (very high correlation);
- between 0.90 and 0.70 (high correlation);
- between 0.70 and 0.40 (moderate correlation);
- between 0.40 and 0.20 (weak correlation);
- between 0.20 and 0.00 (very weak correlation).^19^

## RESULTS

Fifty-three patients were included in the study, 30 of them (56.6%) being males. One patient was excluded because, despite having been diagnosed with the disease, he was oligosymptomatic and did not use any of the medications in study. The median age of patients was 112 (13-231) months. From all patients, 33 (62.3%) lived in cities in the countryside of the State. Table 1 presents other socioeconomic information and data on clinical variables studied. The primary caregivers were the mother and the father of 36 (67.9%) and three (5.7%) patients, respectively. In four cases (7.6%), individuals with other degrees of kinship has this function. Among the 43 caregivers, 12 (27.9%) had incomplete primary education; seven (16.3%) had complete elementary school; 19 (44.2%) had complete high school; and five (11.6%), had complete higher education. Ten patients (18.9%) were mainly responsible for the management of the therapeutic regimen (self-care). The median monthly family income was R$ 1,000 (R$ 300 - R$ 4,000,000), with 19 (38.5%) families receiving up to one minimum wage per month. School dropout (minimum of three months without going to school) was reported for four patients, in all cases due to clinical complications of the disease.

**Table 1:** Clinical and socioeconomic data of the patients.

Socioeconomic profile of patients	n (%)
Social assistance beneficiaries	11 (20.8)
Receiving benefit for patients with chronic diseases	32 (60.4)
School dropout	4 (7.5)
Clinical profile of patients	Mean (SD)
FEV_1_ (%)^a^	84.9% (17.9)
BMI (kg/m^2^)^b^	16.7 (3.0)
Number of hospital admissions for intravenous antibiotic therapy	0.3 (0.6)^c^
Shwachman-Kulczycki Score	85.1 (13.6)

SD: standard deviation; FEV_1_: forced expiratory volume in the first second; BMI: body mass index; ^a^data available only for 35 patients (spirometry is performed only on patients from seven years of age), median FEV_1_ of 85%; ^b^median BMI = 16.08 kg/m^2^; ^c^median number of hospital admissions was zero.

Among patients, 20 (37.7%) reported assisted respiratory physical therapy, of which 45% (n=9) did it only once a week, while the others (55%) did it twice or more times a week. Twenty-three patients (43.4%) were on home respiratory physical therapy, with frequencies ranging from sporadic (n=3; 13%) to daily (n=5; 21.7%); while 17 (73.9%) patients were on home physical therapy twice or more times a week.

The mean OAI was 83.2% (SD=15.3). Table 2 lists the TAI for the different forms of treatment and the main difficulties related to adherence reported by the interviewees. Of these, 27 (50.9%) reported forgetfulness of treatment. Among them, the medications most frequently overlooked were dornase alfa (10 patients - 37%) and vitamin supplementation (25.9%, n=7), while pancreatic enzymes were reported by 66.7% (n=18) as the least frequently forgotten.

**Table 2:** Patients’ adherence rates and main difficulties identified.

Treatment modality (number of patients using them)	Mean TAI (SD)
Dornase alfa (n=39)	86.1% (29.4)
Pancreatic enzymes (n=45)	96.6% (9.6)
Inhaled Tobramycin for Continuous Use (n=5)	78.6% (29.4)
Vitamin Supplements (n=52)	88.1% (28.5)
Nutritional Supplements (n=14)	51.8% (50.4)
Oil addition to food (n=52)	77.0% (32.4)
Nutritional guidelines (n=53)	78.0% (16.6)
Difficulties related to adherence reported by patients	n (%)
No time	7 (3.2)
Too many medications in the therapeutic routine	8 (15.1)
Poor access to medications or supplements	26 (49.1)
No understanding of how to use medications	1 (1.9)
Feeling of embarrassment when using medications in front of other people	11 (20.8)
Reduction of pancreatic enzymes for weight loss	2 (3.8)

TAI: therapeutic adherence index; SD: standard deviation.

Due to changes in the staff composition at the institution, the assessment of patients’ TA by health professionals included only those who entered the outpatient clinic until 2014 (n=39). As not all patients were on psychological counseling, only 29 of them were included in the assessment of this specialty. In the areas of nutrition, pharmacy and pulmonology, a form was not filled in, resulting in a final sample of 38 patients. The mean scores provided by the team were 7.7±1.6 by pulmonology; 7.0±1.0 by physical therapy; 6.9±0.29 by psychology; 6.8±2.4 by nutrition; and 6.3±2.0 by pharmacy.

When assessing the association between TA and sociodemographic and clinical variables, a statistically significant association between satisfactory TA and age below 14 years was found (p=0.021). The evaluation on the association between TA and the other variables, on the other hand, resulted not statistically significant (Table 3). There was a statistically significant positive correlation between the scores of psychologists (p=0.032) and nutritionists (p=0.012), which did not occur in the other areas (Table 4).

**Table 3:** Association between adherence to treatment and clinical/sociodemographic variables.

Variables	Adherence: n (%)		p-value ^a^
	Satisfactory	Unsatisfactory	
Age			
≥14 years	6 (17.6)	9 (47.4)	0.021
<14 years	28 (82.4)	10 (52.6)	
Gender			
Female	16 (47.1)	7 (36.8)	0.472
Male	18 (52.9)	12 (63.2)	
Family income^b^			
<1 minimum wage	2 (5.9)	0	0.333
1-2 minimum wages	21 (61.8)	15 (78.9)	
>2 minimum wages	11 (32.3)	4 (21.1)	
Age at diagnosis			
≤12 months	14 (41.2)	4 (21.1)	0.226
>12 months	20 (58.8)	15 (78.9)	
Schooling			
Incomplete elementary school	7 (23.4)	3 (23.0)	0.154
Complete primary education	4 (13.3)	5 (38.5)	
Complete high school or more	19 (63.3)	5 (38.5)	
Benefits from the Government			
Yes	23 (67.6)	13 (68.4)	0.954
No	11 (32.4)	6 (31.6)	
Poor access to medications			
Yes	15 (44.1)	11 (57.9)	0.336
No	19 (55.9)	8 (42.1)	
Schwachman score			
86-100	17 (50.0)	6 (31.6)	0.448
71-85	12 (35.3)	7 (36.8)	
56-70	4 (11.8)	5 (26.3)	
41-55	1 (2.9)	1 (5.3)	
FEV_1_			
≥80	13 (70.6)	11 (68.8)	0.559
60-79	4 (26.5)	5 (31.2)	
40-60	0	0	
<40	1 (2.9)	0	
BMI or weight/height			
Risk/overweight	6 (17.6)	1 (5.3)	0.261
Eutrophic	27 (79.4)	16 (84.2)	
Thinness	1 (3.0)	2 (10.5)	

FEV_1_: forced expiratory volume in the first second; BMI: body mass index; ^a^chi-square test; ^b^a minimum wage corresponded to R$ 788.00 (U$ 215.31).

**Table 4:** Evaluation of the association between the notes provided by the team’s health professionals, referring to the assessment of patients’ adherence to treatment (n = 38), with self-reported global adherence rates.

Field	**R**	p-value^*a*^
Pharmacy	0.210	0.206
Physical therapy	0.117	0.479
Psychology	0.399	0.032
Nutrition	0.402	0.012
Pneumology	0.162	0.332

^a^Spearman’s correlation.

## DISCUSSION

There was a high rate of global TA (83.2%) in this study, compared to the variations from 31 to 79% described in the literature,^7^ ^,^ ^10^ which may be due to the age group studied. Previous studies have shown better TA in CF among children compared to adolescents.^20^ ^,^ ^21^ Among the possible causes for higher rates of TA in childhood, there would be a lower rate of participation of parents in the administration of treatment during adolescence, a moment of transition in which one’s perceptions about their own disease are established, in addition to several personal, professional and social changes and challenges in which the clinical progression of CF can have a direct impact, thus influencing TA.^14^ ^,^ ^20^ It is essential, therefore, to emphasize the relevance of family participation in the adaptation of adolescents to their therapeutic routine, without compromising the development their individual autonomy. The age at diagnosis, in turn, was not significantly associated with TA, as previously described in the literature.^15^

The present study covered very low-income patients and, even so, high rates of global TA were found. The routine of supplying and dispensing medications, available at the center, is probably an important factor that contributed to this phenomenon. The importance of a multidisciplinary team with close contact with patients should also be emphasized, enabling multiple approaches that promote a better TA. The patient’s solid relationship with the health team as an important facilitator of TA has been previously described.^22^ ^,^ ^23^

In this study, adherence to ETR was the highest (96.6%), in addition to being indicated as the drug category that was less frequently overlooked by participants. These data are in agreement with that described in another Brazilian study, in which 96.3% of the patients were in the group of high adherence to this therapy.^15^ The reported adherence to ERT is within the percentages reported in other studies (27,4 to 96.5%).^9^ ^,^ ^10^ ^,^ ^21^ The greater adherence to ERT is possibly due to steatorrhea and other immediate consequences of not using enzymes.^24^ The perception of signs and symptoms is an important factor in raising awareness of patients for TA. Difficulties regarding this adherence in asymptomatic pediatric patients have already been detected in previous studies.^12^

The adherence to respiratory physical therapy was very low in this research, inferior to data in the literature, which indicate TA rates from 36 to 91% for this treatment modality.^10^ In the present study, low family income and residence in the countryside of the State (62.3% of patients), where the availability of physical therapy services is poor, must have contributed to the low adherence to this modality. Even in the capital, difficulties were reported for regular monitoring by these professionals. Among patients who did not undergo assisted respiratory physical therapy, 72.7% (n=24) reported difficulties in getting monitored by a physical therapist, which reflects the problems of access to the health system. Unfortunately, this center does not provide outpatient physical therapy follow-up; it is only available during outpatient consultations or hospital admissions.

The average scores given by the health professionals ranged from 6.3 to 7.7. Arias Llorente et al., in 2008, also evaluated the opinion of health professionals (nurses, pulmonologists and gastroenterologists) regarding TA of patients, reporting 35.1% (physiotherapy) to 70.4% (digestive tract medications).^21^ In that study, however, the methodology for assessing TA by professionals differed from the one used in the present study, making comparisons difficult, which is a common problem due to the diversity of strategies reported in the literature to classify and analyze TA.^10^

There was a positive association between TA scores provided by psychology and nutrition professionals and the self-reported OAI. Such data possibly stems from aspects intrinsic to the routine of care of these professionals, which can provide more accurate information about the degree of dedication of patients to the proposed therapeutic regime. Nutritional assessment, for example, includes obtaining regular food records. Psychology, in turn, is dedicated to addressing in-depth psychosocial issues critical to TA. However, it is worth mentioning that these correlations, although statistically significant, were weak. On the other hand, there was a positive correlation between the scores attributed by other professionals and TA, although it was not statistically significant, possibly due to the small sample size. Therefore, further studies are needed to assess this issue.

There are several methods of measuring TA available.^25^ Modi et al., In 2006, carried out a study that evaluated adherence through different methods and compared them with electronic monitoring, which would correspond to the most accurate form of investigation, and found that the self-reported approach had very different results in relation to the other methods, overestimating adherence.^26^ Thus, the self-reported method used in the present study may have contributed to the high adherence percentages. It is noteworthy, however, that a previous study, using a more accurate measurement methodology, reported a high rate of adherence in pediatric CF patients, suggesting that TA may be higher among them.^27^ Ball et al., in 2013, investigated adherence to inhaled medications using electronic monitoring in patients aged 11 to 17 years and reported an adherence rate greater than 75% in half of them.^27^ Thus, it is also possible that the high TA observed in this study is not due to the measurement method, but rather to the age group of patients. In addition, the positive correspondence between psychology and nutrition assessments and patients’ self-assessment may indicate greater reliability of the self-reported TA in this study.

We can list the following methodological limitations of the present study:

- the small number of patients studied;
- the age group of the population, in which the most severe clinical manifestations of the disease were not so common, as well as the wide age range of the patients;
- the fact that the study covers a single reference center;
- the self-reported methodology for measuring TA;
- the change in the composition of the service team, which made it difficult to collect the opinion of health professionals regarding the TA.

Thus, additional studies with a larger number of participants are needed, as well as different tools for measuring TA in CF, which has several peculiarities due to the complexity of treatment and the chronic character of the disease.

To conclude, it appears that the overall rate of TA was high, although TA was unsatisfactory in certain therapeutic modalities, such as nutritional guidelines and continuous inhaled tobramycin. Adherence to respiratory physical therapy was very low, in fact lower than previous reports in the literature. There was a satisfactory association between age below 14 years and TA. The average scores attributed to patients’ TA by professionals from the reference center varied little between different areas of health, with the pharmacy providing the lowest average and pneumology, the highest. There was a positive correlation only between TA scores inferred by psychology and nutrition professionals compared to TA self-reported by patients/guardians. The results point to the importance of monitoring TA in chronic diseases, particularly among adolescents.
